# Urinary sodium-to-potassium ratio: a simple and useful indicator of diet quality in population-based studies

**DOI:** 10.1186/s40001-020-00476-5

**Published:** 2021-01-06

**Authors:** Parvin Mirmiran, Zahra Gaeini, Zahra Bahadoran, Asghar Ghasemi, Reza Norouzirad, Maryam Tohidi, Fereidoun Azizi

**Affiliations:** 1 Nutrition and Endocrine Research Center, Research Institute for Endocrine Sciences, Shahid Beheshti University of Medical Sciences, No. 24, Sahid-Erabi St, Yemen St, Chamran Exp, P.O. Box: 19395-4763, Tehran, Iran; 2 Endocrine Physiology Research Center, Research Institute for Endocrine Sciences, Shahid Beheshti University of Medical Sciences, Tehran, Iran; 3 Department of Biochemistry, School of Paramedical Sciences, Dezful University of Medical Sciences, Dezful, Iran; 4 Prevention of Metabolic Disorders Research Center, Research Institute for Endocrine Sciences, Shahid Beheshti University of Medical Sciences, Tehran, Iran; 5 Endocrine Research Center, Research Institute for Endocrine Sciences, Shahid Beheshti University of Medical Sciences, Tehran, Iran

**Keywords:** Sodium, Potassium, Urine, Dietary pattern

## Abstract

**Background:**

Current evidence regarding the prognostic relevance of urinary sodium-to-potassium ratio (Na-to-K ratio), as an indicator of diet quality is limited. This study was conducted to investigate whether urinary Na-to-K ratio could be related to habitual dietary patterns, in a general population.

**Methods:**

This study was conducted in the framework of the Tehran Lipid and Glucose Study (2014–2017) on 1864 adult men and women. Urinary Na and K concentrations were measured in the morning spot urine samples. Dietary intakes of the participants were assessed using a validated 147-item Food Frequency Questionnaire (FFQ) and major dietary patterns were obtained using principal component analysis. Mediterranean dietary pattern and Dietary Approaches to Stop Hypertension (DASH) score, were also calculated. Multivariable-adjusted linear regression was used to indicate association of dietary patterns and urinary Na-to-K ratio.

**Results:**

Mean (± SD) age of participants was 43.7 ± 13.9 years and 47% were men. Mean (± SD) urinary Na, K and the ratio was 139 ± 41.0 and 57.9 ± 18.6 mmol/L, 2.40 ± 0.07, respectively. Higher urinary Na-to-K ratio (> 2.37 vs. < 1.49) was related to lower intakes of vegetables (282 vs. 321 g/day), low-fat dairy (228 vs. 260 g/day) and fruits (440 vs. 370 g/day). Western dietary pattern was related to higher urinary Na-to-K ratio (*β* = 0.06; 95% CI 0.01, 0.16). Traditional dietary pattern, Mediterranean and DASH diet scores were inversely associated with urinary Na-to-K ratio (*β* = − 0.14; 95% CI − 0.24, − 0.11, *β* = − 0.07; 95% CI − 0.09, − 0.01, *β* = − 0.12; 95% CI − 0.05, − 0.02, respectively).

**Conclusions:**

Spot urinary Na-to-K ratio may be used as a simple and inexpensive method to monitor diet quality in population-based epidemiological studies.

## Background

Excessive dietary intakes of sodium (Na) along with insufficient potassium (K) intakes are related to risk of developing cardiometabolic disorders [1, 2]. The ratio of Na to K is now suggested as a more reliable index to assess the risk of cardiovascular diseases (CVD) and CVD-related mortality than either Na or K intake alone [3]. Urinary Na-to-K ratio has been shown as a strong predictor of hypertension (HTN) and cardiovascular diseases (CVD) [4–7]. The ideal Na-to-K ratio of diet still remains a matter of debate, due to controversy about the ideal level of Na and K intakes; the value of ~ 0.5 derived from the World Health Organization (WHO) and US–Canada recommendations (i.e., 2000 mg/day of Na and 3500 mg/day of K and 2300 mg/day Na and 4700 mg/day K, respectively) has been suggested [8, 9], however a ratio of < 1.0 has been identified as a best balance of Na and K intakes for preventing CVD and all-cause mortality [10].

Non-urinary-based dietary assessments methods (e.g., 24-h food recalls, Food Frequency Questionnaire) and measurement of urinary Na and K concentrations, in both spot and 24-h samples, are the most common methods for estimation of dietary Na and K intakes [11, 12]. Although measurement of Na and K in 24-h urine is the gold standard method to estimate their dietary intakes, low feasibility in population-based studies and incomplete collections, resulting in underestimation of NA–K intakes, is the major limitation of the method [13]. In contrast, spot urine sampling has a negligible risk of collection errors and is less burdensome and more cost-effective than 24-h urine sampling, which makes this method more practical, especially for population-based surveys [12]. There are a number of studies suggested spot urinary Na and K as a simple, non-invasive, useful and alternative method to 24-h urine estimation in populations [14, 15].

Current evidence regarding the prognostic relevance of urinary Na-to-K ratio, as an indicator of diet quality in population-based studies is limited. Few observational studies have investigated the association between urinary Na-to-K ratio and dietary patterns [16, 17]. In the current study, we aimed to investigate whether spot urinary Na-to-K ratio could be related to habitual dietary patterns, and how the index can be used as an indicator of diet quality among a general population.

## Methods

### Study population

Current study was conducted within the framework of Tehran Lipid and Glucose Study (TLGS), a prospective study on a representative sample of residents from district 13 of Tehran, to investigate and prevent non-communicable diseases (NCD) [18]. TLGS is a community-based study that was initiated in 1999 with 15,005 individuals, aged ≥ 3 years, and data collection is ongoing every 3 years to assess changes of NCD risks [19]. For the current analysis, we recruited 1864 adult men and women (age ≥ 19 years) from the sixth examination of the TLGS (2014–2017); participants with complete data on spot urinary values (Na, K and creatinine), demographics, anthropometrics, biochemical measurements and dietary intakes were included. Participants who had under- or over-reported of energy intakes (< 800 kcal/day or > 4200 kcal/day, respectively) were excluded from the final analysis.

### Anthropometric and demographic measures

Weight was measured by digital scales (Seca, Hamburg, Germany), height and waist circumference were measured by a tape meter, measurements were reported to the nearest of 100 g and 0.5 cm, respectively. Waist circumference was measured at the level of the umbilicus. Subjects were minimally clothed and without shoes for anthropometric measurements. Body mass index (BMI) was calculated as weight (kg) divided by height in square (m^2^).

Systolic (SBP) and diastolic (DBP) blood pressures were measured using a standard mercury sphygmomanometer calibrated by the Iranian Institute of Standards and Industrial Researches [20]. Blood pressure was measured twice on the right arm of the participants, after a 15-min rest in a sitting position, with at least a 30-s interval between two measurements. Mean of the two measurements was considered as the participant’s blood pressure.

### Biochemical measures

Both blood and spot urine samples were obtained between 7:00 and 9:00 a.m. following overnight fasting. Urinary concentrations of Na and K were measured using flame photometry (Screen lyte, Hospitex Diagnostics, Florence, Italy). Intra- and inter-assay coefficients of variations (CVs) were ≤ 2.8% for Na, and ≤ 4.8% for K.

Fasting plasma glucose (FPG) and triglycerides (TG) levels were determined by the enzymatic colorimetric method, using glucose oxidase and glycerol phosphate oxidase, respectively. High-density lipoprotein cholesterol (HDL-C) was measured by a homogenous method (HDLC Immuno FS). Blood analysis were done using Pars Azmoon kits (Pars Azmoon Inc., Tehran, Iran) and a Selectra 2 auto-analyzer (Vital Scientific, Spankeren, The Netherlands) at the research laboratory of the TLGS. Both inter- and intra-assay coefficients of variations (CVs) were ≤ 5%.

### Dietary assessment

Dietary assessment was done using a validated 147-item Food Frequency Questionnaire (FFQ), intake frequency of each typical food item over the previous year documented on a daily, weekly, or monthly basis in household measures [21]. Since the Iranian Food Composition Table (FCT) has limited data on the nutrient content of raw foods and beverages, the US Department of Agriculture’s (USDA) Food Composition Table was used to analyze foods and beverages for their energy and nutrient contents. For the traditional Iranian foods not available in the USDA table, the Iranian FCT was used as an alternative. Validity and reliability of the FFQ have previously been reported [22].

### Identification of dietary patterns and calculation of dietary scores

To obtain major dietary patterns, the principal component analysis (PCA) with varimax rotation was conducted, based on 18 predefined food groups (i.e., whole grains, refined grains, starched vegetables, non-starched vegetables, fruits, beans, high-fat and low-fat dairy, red meat, poultry, vegetable oil, hydrogenated and animal fat, fast foods, salty snacks, sweet snacks, sweetened beverages, nuts and seeds, tea and coffee). PCA, a posteriori (data-driven) approach, is the most commonly used method to derive dietary patterns; this method is a variable-reducing procedure based on correlation or covariance matrices of the original variables, creating linear combinations (components, factors, or patterns) [23].

We considered eigenvalues > 1, the scree plot and the interpretability of the patterns, and 2 factors were obtained. Although all food groups contributed to the pattern score calculation, food groups with an absolute component loading ≥ 0.30 were selected to describe the pattern. The Kaiser–Meyer–Olkin statistic, a measure of sampling adequacy, was 0.67 (values > 0.6 indicate the usefulness of cluster analysis using our data), and the *P* value for Bartlett’s test of sphericity was < 0.001 supporting the use of cluster analysis as an appropriate procedure. Factor scores were calculated using the sum of intakes of the standardized food groups weighted by their respective factor loadings on each pattern.

To estimate Mediterranean dietary pattern scores, we used an index variable that was composed of 8 Mediterranean food groups. The score was proposed by Trichopoulou et al. [24] and its validity was evaluated in previous studies [25]. In brief, consumption of food groups (vegetables, fruits, legumes, nuts, whole grains, fish and MUFA/SAFA) were scored according to median intake values of the study population (i.e., score 0 and 1 for intakes below and above the median). For total red meat, if the subjects consumed more than median, we assigned score 0 and if they consumed less than median, a score of 1 was assigned. Finally, after adding up the individual component scores, overall Mediterranean dietary pattern score ranged from zero to eight [26].

To estimate DASH score, eight food-derived components including high intakes of fruits, vegetables, nuts, legumes, low-fat dairy, and whole grains, and low intakes of sodium, sweetened beverages, and red and processed meats, were considered. For each of the eight components, all participants were categorized into fifths according to their intakes ranking. For components in which higher intakes are desirable (i.e., fruits, vegetables, nuts, legumes, low-fat dairy, and whole grains), individuals received the maximum score of 5, if their intakes were in the highest quintile. The remaining components (i.e., sodium, sweetened beverages, and red and processed meats) were reversely coded. Intakes of the components between minimum and maximum amounts were scored proportionally. Finally, scores were summed to a total DASH score that ranges from a minimum of 8 o to a maximum of 40 points [27]. This scoring system of DASH diet was used in same population [28, 29], and its validity was assessed elsewhere [30].

### Statistical analyses

General characteristics and dietary intakes of participants were compared across tertiles of urinary Na-to-K ratio, using the one-way ANOVA and Chi-square tests, for dichotomous and continues variables, respectively. To assess potential association of dietary patterns scores with urinary Na-to-K ratio, linear regression models, adjusted for potential confounders were used. To identify the potential confounding variables, univariate regression was used and the variables with *P*_E_ < 0.2 were selected to enter the models. Finally, confounders adjusted in models included sex (men/women), age (year), BMI (kg/m^2^), and total energy intake (kcal/day). All statistical analyses were conducted using the Statistical Package for Social Science (version 20; IBM Corp., Armonk, NY, USA) and *P*-value < 0.05 was considered significant.

## Results

Mean (± SD) age of the study participants was 43.7 ± 13.9 years, and 47% of the participants were men. Mean (± SD) calorie intake of the participants was 2241 ± 680 kcal/day and mean (± SD) BMI was 27.7 ± 5.10 kg/m^2^. Mean (± SD) dietary intake of Na and K were 3498 ± 1681 and 4415 ± 1697 mg/day, respectively. Mean (± SD) urinary Na and K concentrations, and Na-to-K ratio were 139 ± 41.0 and 57.9 ± 18.6 mmol/L, and 2.40 ± 0.07, respectively.

Demographic characteristics, anthropometric, and biochemical values of the participants across tertiles of Na-to-K ratio are shown in Table 1. Dietary intakes of the participants across tertiles of Na-to-K ratio are presented in Table 2. Participants in the highest tertile of urinary Na-to-K ratio, compared to those in the first tertile, had significantly lower consumption of fruits (370 vs. 440 g/day), vegetables (282 vs. 321 g/day), low-fat dairy (228 vs. 261 g/day), nuts and seeds (12.04 vs. 14.7 g/day). Dietary intakes of refined grains and hydrogenated and animal fats were significantly higher in the last tertile of urinary Na-to-K ratio, compared to the first (284 vs. 247 g/day and 17.34 vs. 14.99 g/day, respectively). There was no significant difference in dietary intakes of other food groups across tertiles of urinary Na-to-K ratio.

**Table 1:** Demographic characteristics, anthropometric and biochemical values of participants across tertiles of Na-to-K ratio: Tehran Lipid and Glucose Study

Variables	Tertile 1 (*n* = 622)	Tertile 2 (*n* = 621)	Tertile 3 (*n* = 621)	*P*-value
Urinary Na-to-K ratio				
Range	< 1.49	1.49–2.37	> 2.37	
Median	1.03	1.89	3.20	
Age (years)	43.4 ± 13.8	44.2 ± 13.8	43.3 ± 14.3	0.504
Men (%)	44.7	47.2	49.4	0.255
Body mass index (kg/m^2^)	27.9 ± 5.2	27.8 ± 5.0	27.4 ± 5.0	0.159
Waist circumference (cm)	93.1 ± 12.7	93.2 ± 11.8	92.7 ± 12.3	0.745
Fasting plasma glucose (mg/dL)	96.80 ± 27.50	98.34 ± 32.77	95.71 ± 24.65	0.265
Systolic blood pressure (mmHg)	11.20 ± 1.54	11.27 ± 1.69	11.27 ± 1.62	0.727
Diastolic blood pressure (mmHg)	7.51 ± 0.93	7.59 ± 1.04	7.60 ± 1.06	0.247
TG/HDL ratio	3.61 ± 3.70	3.54 ± 3.72	3.26 ± 2.86	0.168

Data are mean ± SD (unless stated otherwise)

*TG* triglyceride, *HDL* high-density lipoprotein

**Table 2:** Dietary intakes of participants across tertiles of Na-to-K ratio: Tehran Lipid and Glucose Study

Variables	Tertile 1 (*n* = 622)	Tertile 2 (*n* = 621)	Tertile 3 (*n* = 621)	*P*-value
Urinary Na-to-K ratio				
Range	< 1.49	1.49–2.37	> 2.37	
Median	1.03	1.89	3.20	
Energy (kcal/day)	2236 ± 697	2208 ± 678	2276 ± 662	0.209
Fruits (g/day)	440 ± 352	408 ± 338	370 ± 304	0.001
Starched vegetables (g/day)	26.81 ± 25.32	26.89 ± 24.17	25.79 ± 21.91	0.666
Non-starched vegetables (g/day)	322 ± 200	286 ± 164	282 ± 166	< 0.001
Refined grains (g/day)	248 ± 154	264 ± 151	285 ± 165	< 0.001
Whole grains (g/day)	137 ± 95.9	136 ± 104	148 ± 113	0.070
Poultry (g/day)	30.62 ± 27.09	32.80 ± 40.24	29.72 ± 28.82	0.231
Red meat (g/day)	20.58 ± 19.40	21.46 ± 30.62	20.11 ± 17.27	0.586
Low-fat dairy (g/day)	261 ± 188	235 ± 179	228 ± 158	0.003
High-fat dairy (g/day)	111 ± 128	110 ± 123	114 ± 135	0.843
Hydrogenated and animal fat (g/day)	14.99 ± 15.46	15.63 ± 15.22	17.34 ± 17.21	0.029
Vegetable oil (g/day)	10.55 ± 26.58	9.69 ± 9.60	9.00 ± 7.38	0.269
Nuts and seeds (g/day)	14.69 ± 23.12	11.58 ± 13.65	12.04 ± 14.50	0.004
Beans (g/day)	41.99 ± 31.50	42.74 ± 34.79	43.77 ± 36.01	0.654
Sweets (g/day)	51.13 ± 88.90	48.43 ± 51.33	46.59 ± 39.05	0.449
Salty snacks (g/day)	16.12 ± 57.38	14.46 ± 20.38	17.04 ± 22.53	0.471
Fast foods (g/day)	15.94 ± 21.74	14.71 ± 21.82	18.29 ± 33.35	0.051
Tea and coffee (mL/day)	586 ± 511	580 ± 457	575 ± 453	0.918
Sweetened beverages (mL/day)	42.39 ± 66.04	46.37 ± 90.13	47.58 ± 75.44	0.471

Data are mean ± SD

Principal component analysis identified two major dietary patterns, traditional and Western dietary patterns. These dietary patterns explained 21.3% of the total variance in food intake overall (variances of 11.7 and 9.6%, respectively). The traditional dietary pattern was characterized by higher loads of starched and non-starched vegetables, sweets and salty snacks, sweetened beverages, fruits, nuts and seeds, poultry, high-fat dairy, red meat, and beans. The Western dietary pattern had higher loads of sweetened beverages, high-fat dairy, refined grains, fast foods, hydrogenated and animal fats. These patterns explained 21.35% of the total variance in the overall dietary intake (Table 3).

**Table 3:** Component loadings for derived dietary patterns by PCA

Foods groups	Dietary patterns	
	1 (traditional)	2 (Western)
Starched vegetables	0.488	
Non-starched vegetables	0.490	
Salty snacks	0.334	
Sweetened beverages	0.332	0.484
Fruits	0.443	
Nuts and seeds	0.390	
Poultry	0.302	
High-fat dairy	0.321	0.344
Sweet snacks	0.331	
Red meat	0.405	
Beans	0.445	
Refined grains		0.437
Fast foods		0.415
Hydrogenated and animal fat		0.341
Total variance	11.75	9.60

Factors loading < 0.3 are not presented for simplicity. Principal component analysis (PCA) was used to obtain main dietary patterns. The derived dietary patterns explained 21.3% of the total variance in food intake overall (variances of 11.7 and 9.6%, respectively)

Mean (± SD) of urinary Na, K and Na-to-K ratio across tertiles of dietary pattern scores are shown in Table 4. Mean urinary Na concentration, as well as urinary Na-to-K ratio, were significantly lower in participants in the highest tertiles of traditional dietary pattern, DASH and Mediterranean dietary pattern (*P*-value for all < 0.05). However, mean urinary Na concentration and Na-to-K ratio were significantly higher in participants in the highest tertile of Western dietary pattern (*P*-value < 0.05). There were no significant differences between urinary K concentrations across tertiles of dietary patterns.

**Table 4:** Mean (± SD) of urinary Na, K and Na-to-K ratio across tertiles of dietary pattern scores

Dietary patterns	Tertile 1	Tertile 2	Tertile 3	*P-*value
Traditional dietary pattern score				
Urinary Na (mmol/L)	137.6 ± 54.65	130.9 ± 55.59	125.8 ± 55.01	0.001
Urinary K (mmol/L)	73.22 ± 36.32	75.52 ± 37.83	75.72 ± 37.94	0.430
Urinary Na-to-K ratio	2.31 ± 1.43	2.12 ± 1.33	2.00 ± 1.19	0.001
Western dietary pattern score				
Urinary Na (mmol/L)	121.6 ± 53.72	133.2 ± 54.39	139.6 ± 56.23	0.001
Urinary K (mmol/L)	74.70 ± 39.17	73.90 ± 36.39	75.85 ± 36.51	0.658
Urinary Na-to-K ratio	2.02 ± 1.35	2.20 ± 1.35	2.21 ± 1.27	0.018
DASH dietary score				
Urinary Na (mmol/L)	140.5 ± 54.74	131.6 ± 54.90	122.3 ± 54.77	0.001
Urinary K (mmol/L)	74.18 ± 36.16	75.04 ± 36.67	75.18 ± 39.24	0.881
Urinary Na-to-K ratio	2.33 ± 1.43	2.11 ± 1.28	2.00 ± 1.24	0.001
Mediterranean dietary score				
Urinary Na (mmol/L)	136.5 ± 54.58	128.0 ± 54.79	128.2 ± 55.94	0.007
Urinary K (mmol/L)	75.15 ± 38.79	75.04 ± 37.22	74.31 ± 36.60	0.905
Urinary Na-to-K ratio	2.25 ± 1.41	2.08 ± 1.31	2.07 ± 1.25	0.016

Data are mean ± SD

Associations between dietary pattern scores and urinary Na-to-K ratio are shown in Table 5. After adjustment for confounding variables, traditional dietary pattern (*β* = − 0.14; 95% CI − 0.24, − 0.11), DASH (*β* = − 0.12; 95% CI − 0.05, − 0.02) and Mediterranean dietary pattern (*β* = − 0.07; 95% CI − 0.09, − 0.01) were inversely associated with urinary Na-to-K ratio. In contrast, Western dietary pattern was positively associated with urinary Na-to-K ratio (*β* = 0.06; 95% CI 0.01, 0.16).

**Table 5:** Multivariable association between dietary patterns scores and spot urinary Na-to-K ratio

Dietary patterns scores	Standardized *β* coefficient	95% confidence interval for *β*	*P* value
Traditional dietary pattern			
Crude	− 0.12	− 0.22, − 0.10	0.001
Adjusted^a^	− 0.14	− 0.24, − 0.11	0.001
Western dietary pattern			
Crude	0.06	0.02, 0.14	0.008
Adjusted^a^	0.06	0.01, 0.16	0.021
DASH dietary score			
Crude	− 0.11	− 0.05, − 0.02	0.001
Adjusted^a^	− 0.12	− 0.05, − 0.02	0.001
Mediterranean dietary score			
Crude	− 0.06	− 0.08, − 0.01	0.013
Adjusted^a^	− 0.07	− 0.09, − 0.01	0.009

Linear regression models were used

^a^Adjusted for age, sex, body mass index, energy intake

## Discussion

In the current cross-sectional population-based study, traditional dietary pattern (loading for vegetables, fruits, sweets and salty snacks, sweetened beverages, nuts and seeds, poultry, high-fat dairy, red meat and beans), as well as DASH and Mediterranean dietary pattern were negatively related to urinary Na-to-K ratio. Western dietary pattern, with higher loads of sweetened beverages, high-fat dairy, refined grains, fast foods, hydrogenated and animal fats, was significantly associated with higher urinary Na-to-K ratio. To the best of our knowledge, this is the first study used a comprehensive approach to address potential application of spot urinary Na-to-K ratio, as a simple method to assess diet quality, in the framework of a population-based study.

In our study, healthy dietary patterns identified as Mediterranean and DASH diet, were related to a lower urinary Na-to-K ratio, as a risk factor of cardiometabolic disorders. Our findings are in line with those of previous studies showed that the Japanese dietary pattern, heavily loaded for fish and vegetables, was positively associated with urinary K concentration [16], and studies reported a significant inverse association between “nuts, seeds, fruits and fish” dietary pattern with urinary Na-to-K ratio [17]. Subjects with a higher Mediterranean dietary pattern score had better nutrient profiles, with lower sodium and higher potassium intakes [31]; similarly, the DASH diet provides high amounts of potassium and a restricted amount of sodium (~ 1500 mg/day) that results in a urinary Na-to-K ratio less than 1 [32, 33].

An inverse association we found between Western dietary pattern and urinary Na-to-K ratio is in line with previous findings indicated that the “noodle dietary pattern” [16], and “snacks–fast food–soft drinks” pattern [34] were related with high urinary Na concentrations.

Mean urinary Na-to-K ratio in our population was 2.40 (< 1.39 in the first and > 2.37 in the third tertile); WHO recommends a corrected-value of 1.33 for urinary Na-to-K ratio, that refers to a Na intake less than 2000 mg/day and K intake more than 3500 mg/day (< 1 mmol/mmol or 0.59 g/g) [35, 36]. A wide range of urinary Na-to-K ratio were reported; The Trials of Hypertension Prevention (TOHP), reported a mean value of 2.88 and 2.97 for men and women, respectively, with a higher value in Black compared to White people (3.43 vs. 2.83) [6]. Mean 24-h urinary Na-to-K ratio ranged from 0.01 in Brazil to 7.58 in China; in Asian and Western populations this value was reported approximately in a range of 3–5 [4, 14].

Although measurement of 24-h urinary Na and K is considered as the gold standard method for assessment of their dietary intakes, it is difficult and rather inconvenient for population-based studies to collect 24-h urine without any loss of urine; reports show that the rate of unsuccessful collection of 24 h urine samples is about 40% [15]. Furthermore, much attention has been focused on estimating Na and K intakes using the ‘spot’ urine sample [37], as collection time of the spot urine samples has no restriction [38, 39]. There are a number of confirmed formulas to estimate 24-h urine Na-to-K ratio, using spot urine values of Na and K, in some cases using creatinine values [15, 37]. Pearson correlation coefficients of 24-h urinary Na-to-K ratio and spot urine Na-to-K ratios across the Western/Asian populations were *r* = 0.88 to 0.96 in subgroups categorized by sex and age; moreover, these correlations were *r* = 0.96 and *r* = 0.69 in analyses across populations and individuals, respectively [14], indicating that spot urine Na-to-K ratio could be a useful, low-burden alternative method to 24-h urine estimation in populations, for comparing different populations, as well as indicating annual trends of a particular population; it may not however be suitable for estimating an individual’s 24-h urine excretions [14, 15]. A benefit of the spot urine Na-to-K ratio (compared to Na or K per se) is that no conversion into 24-h excretion values is needed, a factor that usually results in imprecision [12].

The strengths of the present study include its relatively large sample size, use of a validated comprehensive FFQ to assess dietary intakes, and evaluating the association between four dietary patterns and Na-to-K ratio, simultaneously. Although spot urine sample is a simple and low-burden method to estimate 24-h urine concentrations, it has some limitations due to the potential inter- and intra-individual variations of urinary excretions of Na and K, and effect of time of sampling; using spot urine samples may be limited due to circadian rhythms of Na excretion [12].

Taken together, our findings indicates that spot urinary Na-to-K ratio may be used as a simple, non-invasive and inexpensive method to monitor diet quality, especially in population-based studies. The urinary Na-to-K ratio may also be used to track adherence of dietary recommendations in nutrition clinical trials, in which changes of dietary patterns of the participants during the study period is targeted. However, future studies are needed to approve the performance of spot urine Na-to-K ratio as a reliable method of assessing diet quality.

## Data Availability

Not applicable.

## Abbreviations

TLGS: Tehran Lipid and Glucose Study
FFQ: Food Frequency Questionnaire
BMI: Body mass index
SBP: Systolic blood pressure
DBP: Diastolic blood pressure
FPG: Fasting plasma glucose
HDL: High-density lipoprotein
TG: Triglyceride
CI: Confidence interval
